# Use of Radiology, D-Dimer, and Mean Platelet Volume Combination as a Prognostic Marker in Hospitalized Coronavirus Disease-19 Patients

**DOI:** 10.3389/fmed.2021.788551

**Published:** 2022-02-03

**Authors:** Nagihan Durmus Kocak, Ozlem Oruc, Sibel Boga, Cem Acar, Murat Kavas, Selma Aydogan Eroglu, Baran Gundogus, Ozlem Sogukpinar, Sumeyye Bekir, Aysem Askim Oztin Guven, Makbule Ozlem Akbay, Sibel Arinc, Dildar Duman, Huriye Berk Takir, Feride Yaman, Fatma Ozbaki, Esin Sonkaya, Esra Usta Bulbul, Dilem Anil Tokyay, Lale Dagyildizi, Ulku Aka Akturk, Selahattin Oztas, Dilek Ernam, Gokay Gungor, Nalan Adiguzel, Tekin Yildiz, Ozlem Yazicioglu Mocin, Hakan Gunen, Reyhan Yildiz, Tulin Sevim, Tulay Torun

**Affiliations:** ^1^University of Health Sciences Sancaktepe Martyr Prof. Dr. Ilhan Varank Training and Research Hospital, Istanbul, Turkey; ^2^University of Health Sciences Sureyyapasa Chest Diseases and Thoracic Surgery Training and Research Hospital, Istanbul, Turkey; ^3^Goztepe Medical Park Hospital, Istanbul, Turkey; ^4^Department of Chest Diseases Immunology and Allergy Diseases Science, Faculty of Medicine, Ankara, Turkey

**Keywords:** biomarkers, COVID-19, duration of therapy, hospitalization, mean platelet volume, prognosis, thrombosis, length of stay

## Abstract

**Introduction:**

The search for biomarkers that could help in predicting disease prognosis in the Coronavirus Disease-2019 (COVID-19) outbreak is still high on the agenda.

**Objective:**

To find out the efficacy of D-dimer and mean platelet volume (MPV) combination as a prognostic marker in hospitalized COVID-19 patients with bilateral infiltration.

**Materials and Methods:**

Study design: Retrospective observational cohort. Patients who were presented to our hospital between March 16, 2020 and June 07, 2020 were reviewed retrospectively. The primary outcome of the study was specified as the need for intensive care, while the secondary outcomes were duration of treatment and hospitalization. Receiver operator curve (ROC) analyzes were carried out to assess the efficacy of D-dimer and MPV parameters as prognostic markers.

**Results:**

Between the mentioned dates, 575 of 1,564 patients were found to be compatible with COVID-19, and the number of patients who were included in the study was 306. The number of patients who developed the need for intensive care was 40 (13.1%). For serum D-dimer levels in assessing the need for intensive care, the area under the curve (AUC) was found to be 0.707 (95% CI: 0.620–0.794). The AUC for MPV was 0.694 (95% CI: 0.585–0.803), when D-dimer was ≥1.0 mg/L. When patients with a D-dimer level of ≥1.0 mg/L were divided into two groups considering the MPV cut-off value as 8.1, the rate of intensive care transport was found to be significantly higher in patients with an MPV of ≥8.1 fL compared to those with an MPV of <8.1 fL (32.6 vs. 16.0%, *p* = 0.043). For the prognostic efficacy of the combination of D-dimer ≥ 1.0 mg/L and MPV ≥ 8.1 fL in determining the need for intensive care, following values were determined: sensitivity: 57.7%, specificity: 70.8%, positive predictive value (PPV): 32.0%, negative predictive value (NPV): 84.0%, and accuracy: 63.0%. When D-dimer was ≥1.0, the median duration of treatment in MPV <8.1 and ≥8.1 groups was 5.0 [interquartile range (IQR): 5.0–10.0] days for both groups (*p* = 0.64). The median length of hospital stay (LOS) was 7.0 (IQR: 5.0–10.5) days in the MPV <8.1 group, while it was 8.5 (IQR: 5.0–16.3) days in the MPV ≥ 8.1 group (*p* = 0.17).

**Conclusion:**

In COVID-19 patients with a serum D-dimer level of at least 1.0 mg/L and radiological bilateral infiltration at hospitalization, if the MPV value is ≥8.1, we could predict the need for intensive care with moderate efficacy and a relatively high negative predictive value. However, no correlation could be found between this combined marker and the duration of treatment and the LOS.

## Introduction

According to Chinese officials, Severe Acute Respiratory Syndrome-Coronavirus-2 (SARS-CoV-2), a member of the coronavirus family, spread rapidly across the world from the city of Wuhan, China, at the end of December 2019, and this situation was declared a pandemic by the WHO. The disease caused by SARS-CoV-2 has been named Coronavirus Disease-19 (COVID-19). COVID-19 can affect many organs, such as the brain, kidney, and liver, particularly, the lungs (1, 2).

D-dimer is often increased in severe disease, and present data suggest that this situation is associated with hypercoagulation. Prolonged bed rest in patients also probably increases the risk of venous thromboembolism. Based on the published autopsy results, findings that are showing the disease may cause multi-organ thrombosis have been reported. Numerous micro thrombosis and developing organ ischemia are considered to be one of the key factors associated with mortality (3–5). Upon reviewing the studies conducted since the beginning of the pandemic, it was found that a decrease in the number of leukocytes, lymphocytes, eosinophils, and platelets and an increase in the neutrophil/lymphocyte ratio were reported in patients with COVID-19 (6). In a meta-analysis, low platelet count was found to be associated with severe disease and mortality (7). It is considered that there is a relationship between inflammation and susceptibility to thrombosis and platelet activation, and various platelet markers, such as mean platelet volume (MPV), are being investigated for this purpose. It is well known that younger platelets are larger, contain more granules, and have a higher thrombogenic potential. Hence, it is thought that MPV could be more valuable than platelet count in terms of reflecting platelet functions. Several studies have demonstrated the relationship between coronary artery disease (CAD) risk factors, renal failure, the presence of acute myocardial infarction, and increased MPV levels (8–10). In studies on its use in the diagnosis of acute pulmonary embolism (PE), it has been revealed that cut-off values, such as 8.55 or 8.60 fL, could be helpful in the early diagnosis (11, 12). Moreover, when investigated as a prognostic marker in patients with acute PE, MPV values were determined to be higher in patients with adverse cardiovascular events, thrombolysis, or surgical embolectomy (13). There are many studies in the literature in which MPV is used as a prognostic marker in the evaluation of thrombotic processes in various clinical conditions, both in diagnosis and in follow-up.

In this disease, where our knowledge and experience are relatively limited, we also observed thrombocytopenia in some patients during clinical follow-ups. We are of the opinion that determination of laboratory parameters indicating poor prognosis and clinical and radiological findings suggestive of severe pneumonia could provide guidance for early recognition of these cases and initiation of effective treatment as soon as possible. Thus, we planned to investigate the efficacy of this combination as a prognostic indicator in hospitalized patients with COVID-19 by excluding patients diagnosed with embolism and specifying cut-off values for serum D-dimer and MPV levels in patients with bilateral infiltration. The primary outcome of the study was determined as the need for intensive care at follow-up, while the secondary outcomes were determined as treatment and length of hospital stay (LOS).

## Materials and Methods

### Study Design: Retrospective Observational Cohort

Data of the patients, who applied to our hospital between March 16, 2020 and June 07, 2020 and underwent thoracic CT and/or CT-angiography by entering diagnosis codes Z03.9 (observation for suspected disease or condition, unspecified), Z03.8 (observation for other suspected diseases and conditions), and U07.3 (COVID-19), were retrieved from the hospital automation system. The files of the patients were reviewed retrospectively and assessed in terms of their eligibility for the study.

### Patient Selection

Of all the patients who presented with the suspicion of COVID-19 and had CT and/or CT-angiography for the diagnosis, those who had inclusion criteria compatible with our study protocol were evaluated for eligibility. Inclusion criteria were defined as:

1. Based on international criteria to perform diagnosis of COVID-19, patients with positive PCR for coronavirus in nasopharyngeal (NP) and/or nasal swab samples,
2. Patients with negative PCR test, clinically and radiologically (the presence of typical radiological findings for COVID-19: bilateral and peripheral ground-glass opacities and/or alveolar consolidations), diagnosed as COVID-19 and started treatment,
3. Patients without embolism in angiography, if there is clinical suspicion of PE and pulmonary CT-angiography has been performed,
4. Patients with bilateral pneumonia on tomography,
5. The patients whose D-dimer levels were measured were included in the study.

On the other hand, patients with negative PCR and/or CT findings incompatible with COVID-19 in tests performed with suspicion of COVID-19, patients aged under 18, pregnant patients, patients with PE in CT-angiography performed together with the presence of clinical suspicion, those without parenchymal infiltration on CT or unilateral infiltration, patients with unstudied D-dimer levels, patients hospitalized directly in the intensive care unit (ICU), and outpatients were excluded from the study.

### Outcomes (Prognostic Markers)

Primary outcome: Presence of need for ICU transfer, secondary outcomes, i.e., duration of treatment and LOS.

### Independent Variables

Demographic characteristics, symptoms, comorbidities (hypertension, CAD, diabetes mellitus, cardiac failure, chronic obstructive pulmonary disease, asthma, and malignancy), hemogram parameters at admission, serum C-reactive protein (CRP, normal range: 0–5 mg/dl), Ferritin (normal range: 30–400 ng/ml), D-dimer (normal range: 0–0.55 mg/L), Lactate Dehydrogenase (LDH) levels, PCR test result, the severity of pneumonia, duration of COVID-specific treatment (days), and duration of hospitalization (days).

Pneumonia severity was defined based on the Adult Patient Treatment Algorithm of the Ministry of Health. Accordingly, mild-to-moderate pneumonia: patients with symptoms, such as fever, muscle/joint pains, cough, and sore throat, a respiratory rate of <30/min, percent saturation of oxygen (SpO_2_) level of >90% in room air, and those with mild-moderate pneumonia finding on chest X-ray or tomography, were considered to be with mild-to-moderate pneumonia. Severe pneumonia: patients with symptoms, such as fever, muscle/joint pains, cough, and sore throat, with tachypnea (≥30/min), a SpO_2_ level of ≤ 90% in room air, and bilateral diffuse pneumonia findings on chest X-ray or tomography, were considered to be with severe pneumonia.

### Statistical Analysis

Demographic, clinical, laboratory, and radiological characteristics of the patients were recorded. Chi-square test was used to compare the categorical variables of patients with and without intensive care, while parametric (independent samples *t*-test) and non-parametric (Mann-Whitney U test) tests were used to compare continuous variables, depending on the type of distribution. Receiver Operating Characteristics (ROC) analyzes were conducted to assess the efficacy of D-dimer and MPV parameters as prognostic markers. The statistical significance level was considered to be *p* ≤ 0.05.

### Ethics

The study protocol was prepared in accordance with the Declaration of Helsinki and Good Clinical Practices, the permission of the Ministry of Health was obtained before starting the study and it was approved by the Ethics Committee of the University of Health Sciences Sureyyapasa Chest Diseases and Thoracic Surgery Training and Research Hospital (Date: 04.06.2020, Protocol code: 116.2017.161).

## Results

**Figure 1:**
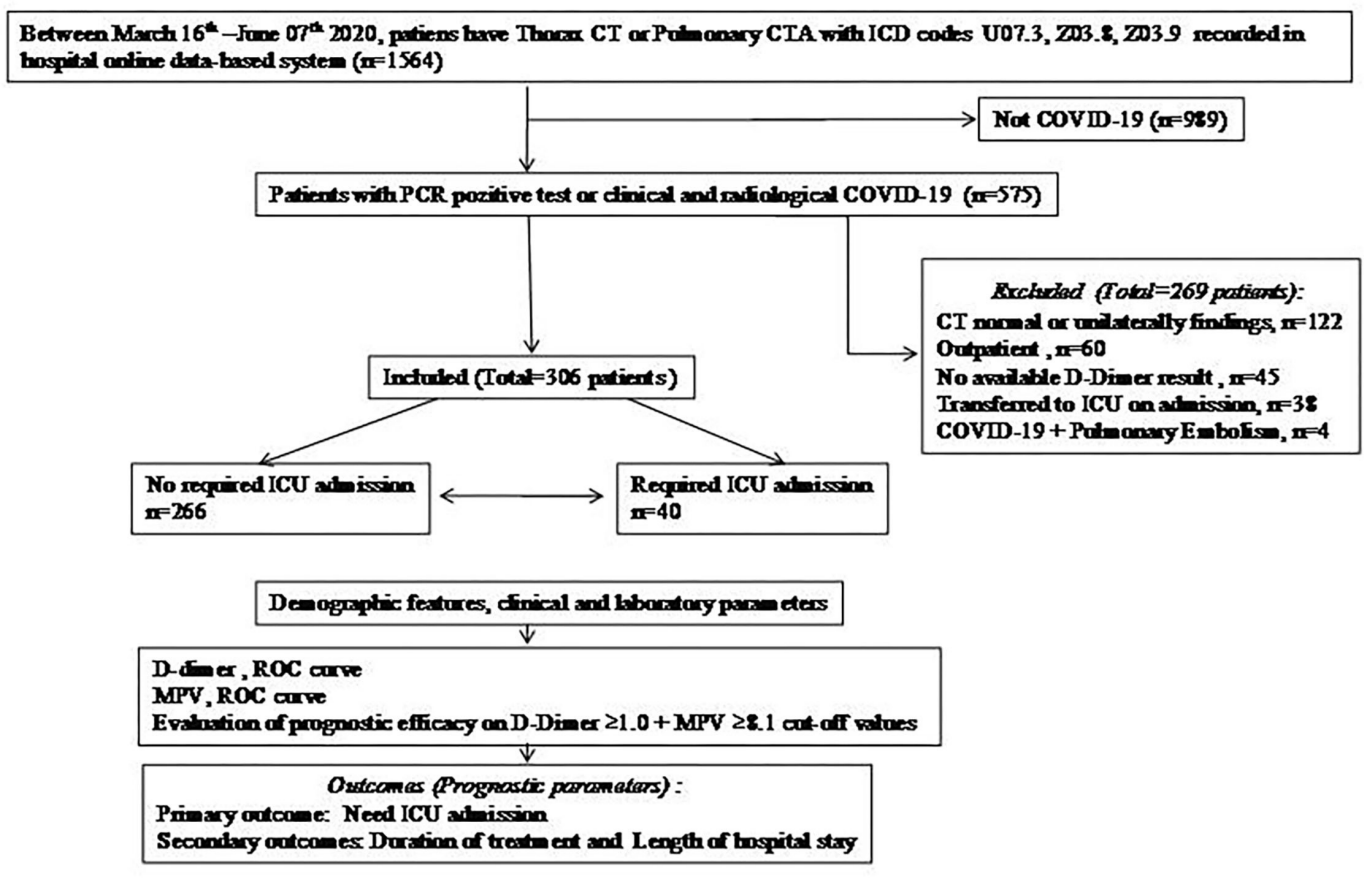
Flow chart. COVID-19, Coronavirus disease-19; CT, computed tomography; CTA, computed tomography angiography; ICU, intensive care unit; MPV, mean platelet volume; PCR, polymerase chain reaction; ROC, receiver operating characteristics.

The file data of a total of 1,564 patients who underwent thoracic CT with the diagnosis codes Z03.9, Z03.8, and U07.3 between the mentioned dates were analyzed and 575 were found to be compatible with COVID. The number of patients who met the inclusion criteria according to the study protocol was 306 (Figure 1).

The primary endpoint of the study, the need for ICU transfer, was developed in 40 (13.1%) of the 306 patients. Comparisons of the patients in terms of the demographic, clinical, and laboratory parameters at the time of admission are presented in Tables 1, 2. Accordingly, no difference was found between the patients who did not need intensive care and those who did, in terms of mean age (53.0 ± 15.8 vs. 57.3 ± 15.4, *p* = 0.11). A total of 180 of the patients were men (58.8%), and the need for intensive care was found to be significantly higher in men (*p* = 0.001). Dyspnea, cough, fever, and malaise were the most common symptoms, and the rate of dyspnea (*p* = 0.027), cough (*p* = 0.015), and diarrhea (*p* = 0.030) were found to be higher in patients who were transferred to the ICU. No difference was determined between the groups in terms of the presence of at least one comorbidity, whereas the rate of CAD was higher in patients who needed intensive care (*p* = 0.010). One hundred seventy-four (56.9%) of all cases were PCR positive and 132 (43.1%) were negative. No correlation was found between PCR results and ICU transfer. When the patients were categorized based on the severity of pneumonia at hospitalization, it was observed that severe cases were significantly higher in those who needed ICU admission during follow-up (*p* = 0.000). When the basic laboratory parameters measured at the time of admission were examined, the lymphocyte count (*p* = 0.000), percentage (*p* = 0.000), and platelet (*p* = 0.037) counts were significantly lower in the patients who were transferred to the ICU, whereas MPV (*p* = 0.015), LDH (*p* = 0.000), CRP (*p* = 0.000), Ferritin (*p* = 0.000), and D-dimer (*p* = 0.000) levels were found to be significantly higher. The median duration of treatment (5.0 vs. 9.0 days, *p* = 0.000) and duration of hospitalization (6.0 vs. 13.5 days, *p* = 0.000) were also longer in patients who needed intensive care.

**Table 1:** Comparison of patients according to intensive care unit transfer with demographic and clinical features on admission.

**Characteristics**	**ICU admission** **not required** **(*n* = 266)**	**ICU admission** **required** **(*n* = 40)**	***p***
Age (mean ± SD)	53.0 ± 15.8	57.3 ± 15.4	0.11
**Gender (** ***n*** **, %)**			
Female	119 (94.4)	7 (5.6)	**0.001**
Male	147 (81.7)	33 (18.3)	
**Symptoms (** ***n*** **, %)**			
Dyspnea	115 (43.2)	25 (62.5)	**0.027**
Cough	151 (56.8)	31 (77.5)	**0.015**
Sputum	39 (14.7)	8 (20.0)	0.36
Fever	119 (44.7)	24 (60.0)	0.09
Sore throat	27 (10.2)	1 (2.5)	0.15
Malaise	104 (39.1)	20 (50.0)	0.23
Nausea	45 (16.9)	12 (30.0)	0.08
Headache	22 (8.3)	5 (12.5)	0.37
Diarrhea	18 (6.8)	7 (17.5)	**0.030**
Anosmia	12 (4.5)	2 (5.0)	1.00
Presence of comorbidity (*n*, %)	166 (62.4)	26 (65.0)	0.86
Hypertansion	79 (29.7)	17 (42.5)	0.14
Coronary artery disease	35 (13.2)	12 (30.0)	**0.010**
Diabetes mellitus	51 (19.2)	10 (25.0)	0.39
Cardiac failure	9 (3.4)	3 (7.5)	0.19
COPD	26 (9.8)	1 (2.5)	0.23
Asthma	42 (15.8)	5 (12.5)	0.81
Malignancy	9 (3.4)	4 (10.0)	0.08
**PCR (** ***n*** **, %)**			
Positive	147 (55.3)	27 (67.5)	0.23
Negative	119 (44.7)	13 (32.5)	
**Severity of pneumonia (** ***n*** **, %)**			
Mild-moderate	237 (97.9)	5 (2.1)	**0.000**
Severe	29 (45.3)	35 (54.7)	

*COPD, chronic obstructive pulmonary disease; ICU, intensive care unit; PCR, polymerase chain reaction; SD, standard deviation. Significant p values were shown as bold characters*.

**Table 2:** Comparison of patients according to the basal laboratory findings on admission, duration of treatment, and length of hospital stay.

**Characteristics**	**ICU admission** **not required** **(*n* = 266)**	**ICU admission** **required** **(*n* = 40)**	***p***
White blood cell count (median, IQR), 10^3^ μl	6.2 (4.7–8.0)	6.8 (4.9–9.0)	0.07
Lymphocyte count (median, IQR),10^3^μl	1.4 (1.0–1.9)	1.0 (6.4–1.5)	**0.000**
Lymphocyte percentage (median, IQR), %	24.2 (18.3–31.6)	15.0 (9.0–20.1)	**0.000**
Red blood cell count (mean ± SD), 10^6^ μl	4.7 ± 0.6	4.7 ± 0.5	0.64
Hemoglobin (mean ± SD), g/dl	13.0 ± 1.7	13.5 ± 1.7	0.09
Hematocrit (median, IQR), %	39.5 (36.5–43.3)	41.5 (38.5–44.3)	0.07
Platelet count (median, IQR), 10^3^ μl	271.5 (202.5–308.5)	236.5 (158.5–279.0)	**0.037**
Mean platelet volume (median ± SD), fL	8.10 ± 0.40	8.50 ± 1.20	**0.015**
Lactate dehydrogenase (median, IQR), U/L	231.0 (187.0–287.0)	329.0 (260.0–405.0)	**0.000**
C-reactive protein (median, IQR), mg/dl	33.9 (5.6–48.5)	90.4 (45.5–143.0)	**0.000**
Ferritin (median, IQR), ng/ml	148.1 (64.9–317.7)	443.0 (216.0–884.9)	**0.000**
D-Dimer (median, IQR), mg/L	0.6 (0.4–1.2)	1.3 (0.6–2.4)	**0.000**
Duration of treatment (median, IQR), days	5.0 (5.0–6.0)	9.0 (5.0–10.0)	**0.000**
Length of hospital stay (median, IQR), days	6.0 (5.0–8.0)	13.5 (9.0–23.3)	**0.000**

*ICU, intensive care unit; IQR, interquartile range; SD, standard deviation. Significant p values were shown as bold characters*.

**Figure 2:**
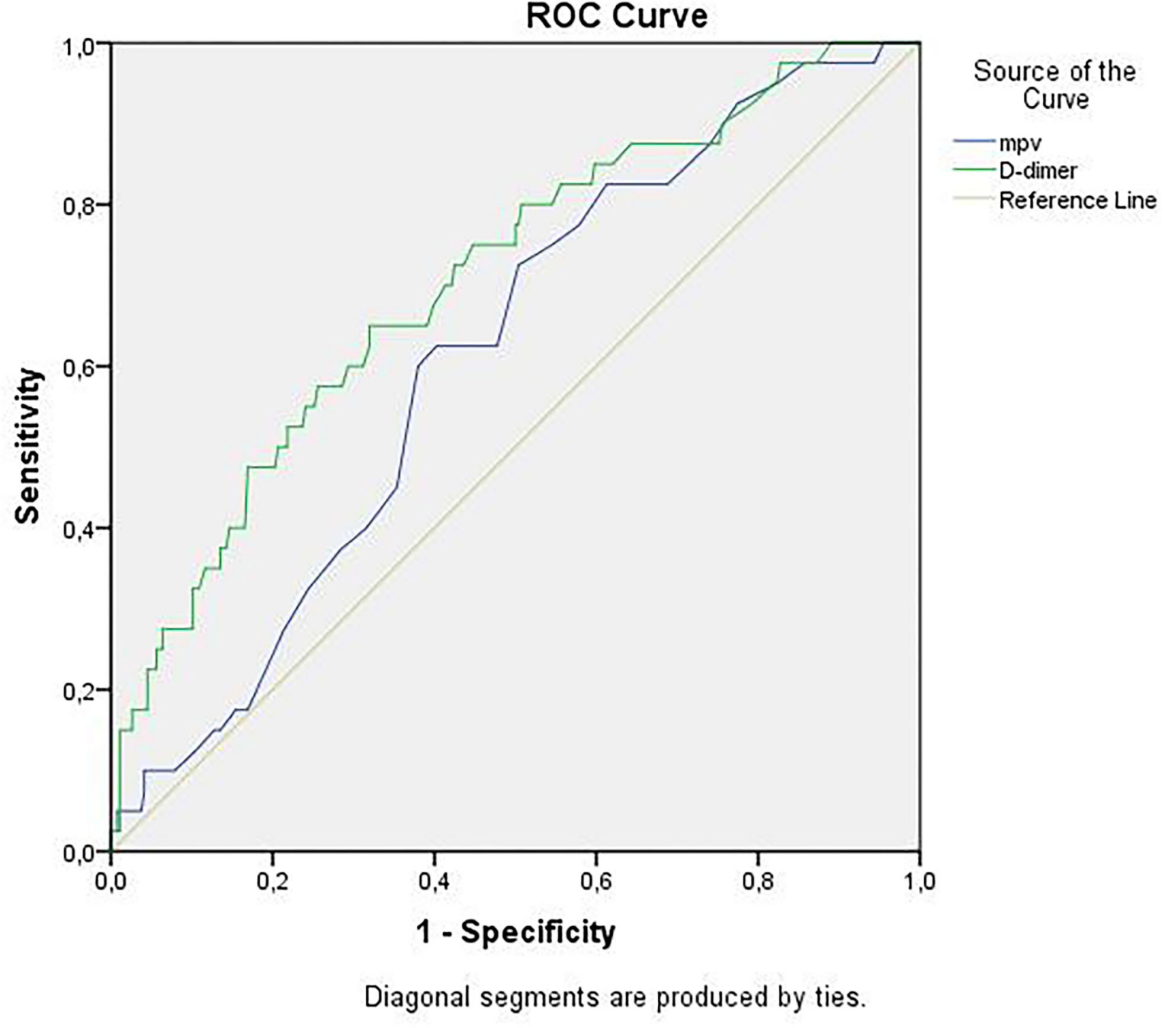
ROC curves of D-dimer and mean platelet volume for intensive care unit transfer state. ROC, receiver operating characteristics.

**Figure 3:**
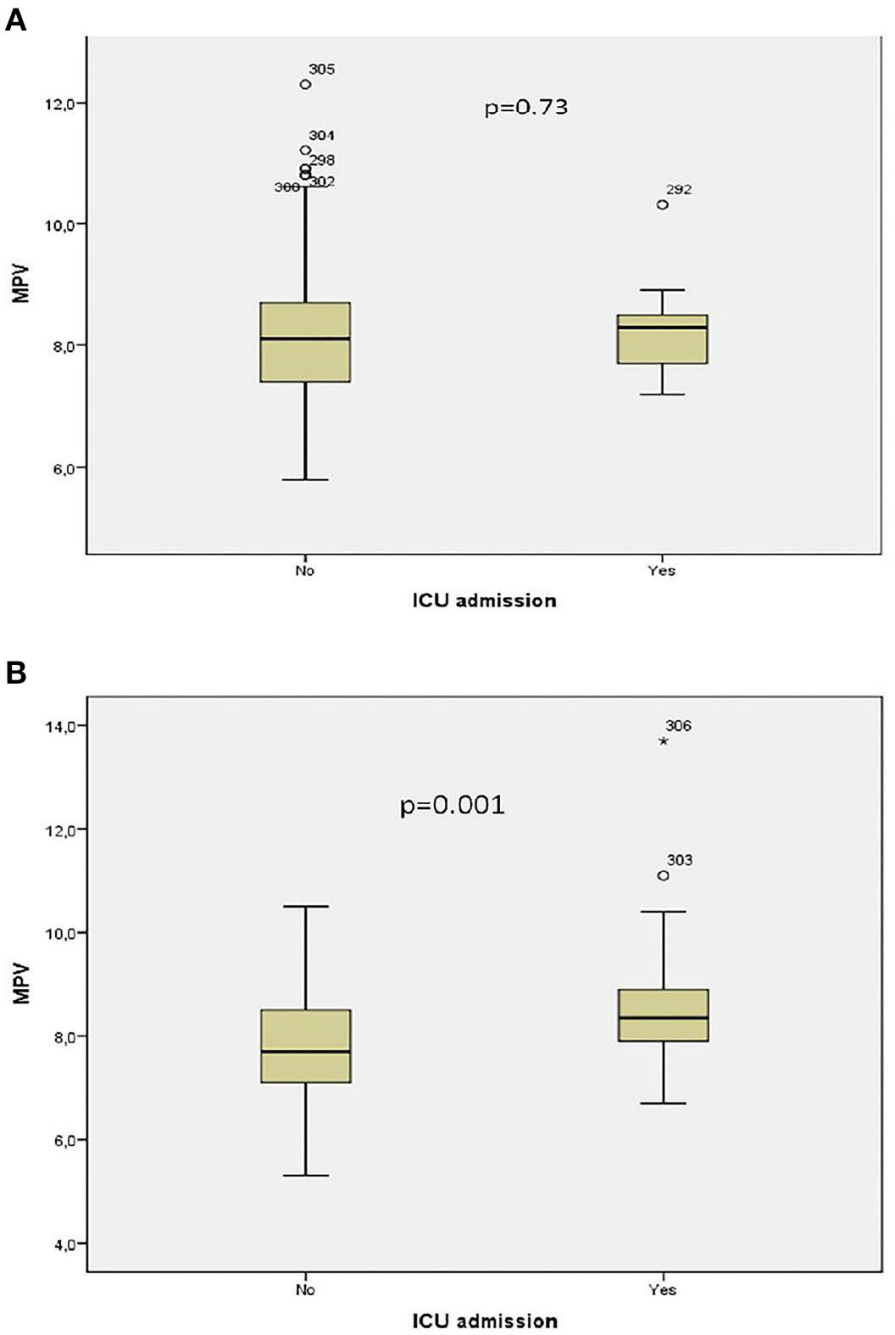
**(A)** Association between intensive care unit transfer and mean platelet volume levels when D-dimer <1.0 mg/L. **(B)** Association between intensive care unit transfer and mean platelet volume levels when D-dimer ≥ 1.0 mg/L. * means that patient no 306 has extreme score for MPV value.

**Figure 4:**
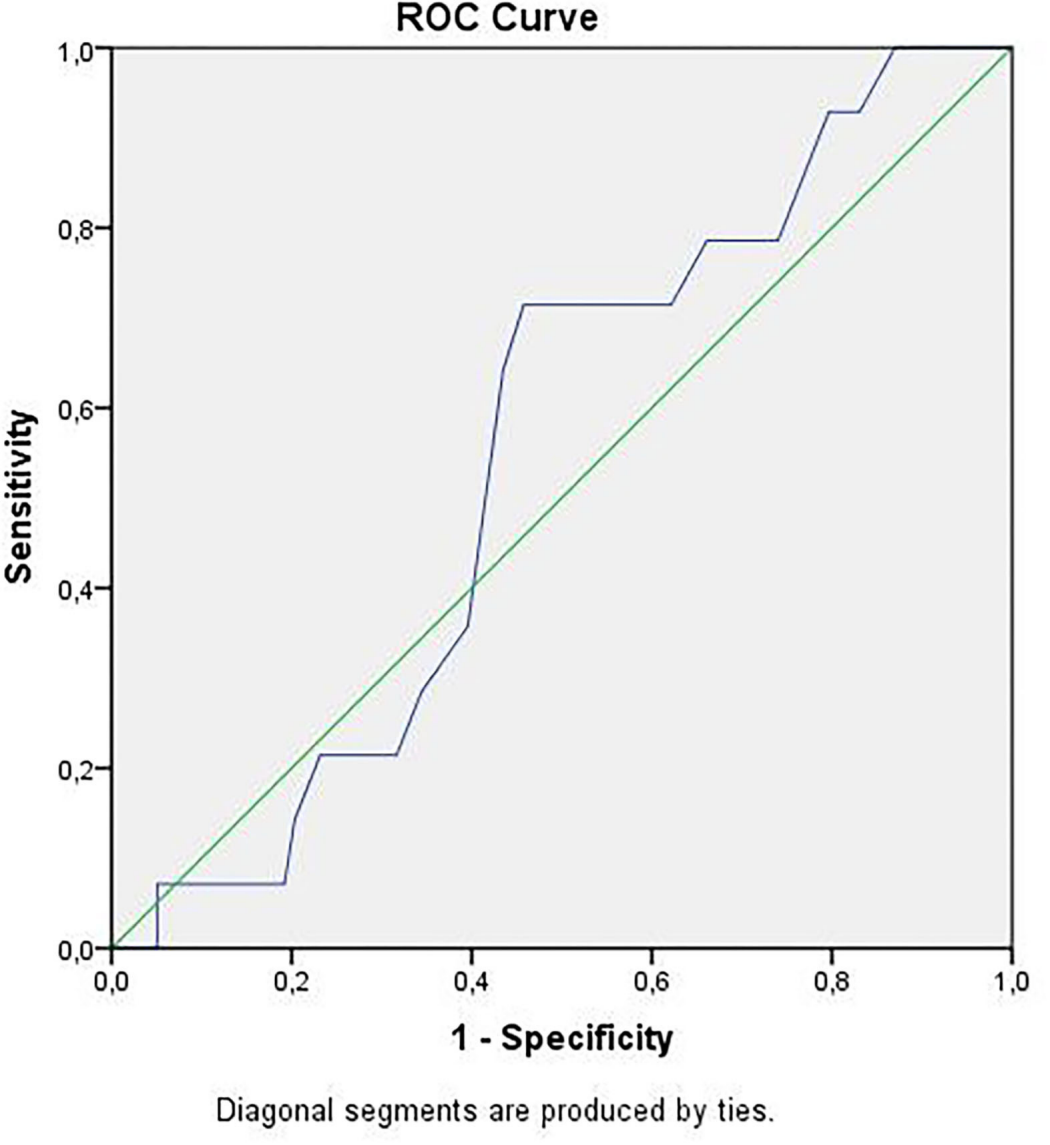
ROC curve of mean platelet volume for intensive care unit transfer state when D-dimer <1.0 mg/L. ROC, receiver operating characteristics.

**Figure 5:**
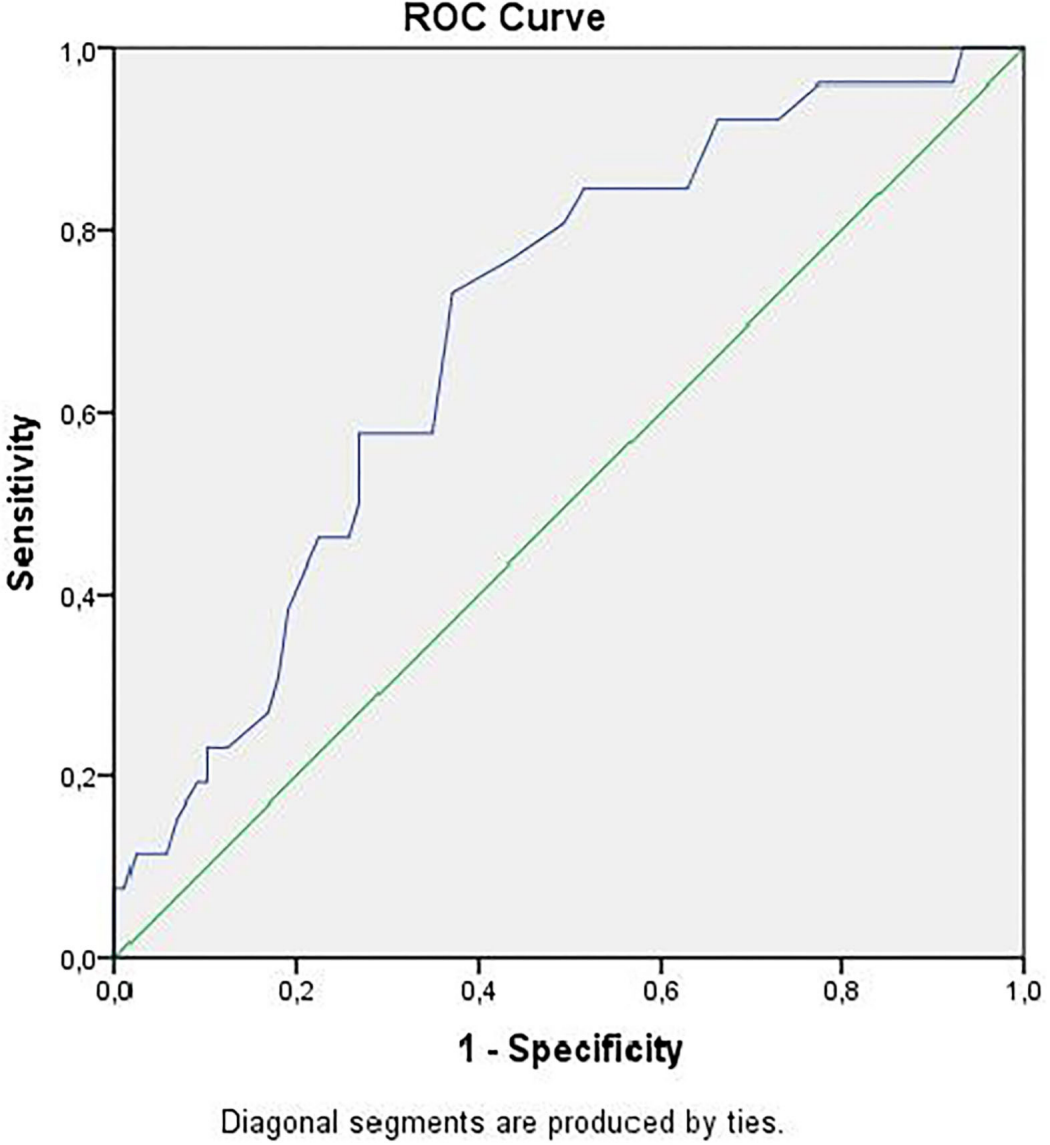
ROC curve of mean platelet volume for intensive care unit transfer state when D-dimer ≥ 1.0 mg/L. ROC, receiver operating characteristics.

Receiver operator curve analysis that was conducted to determine the efficacy of serum D-dimer (area under the curve: AUC: 0.707, 95% CI: 0.620–0.794) and MPV (AUC: 0.612, 95% CI: 0.527–0.696) parameters in assessing the need for ICU transfer is presented in Figure 2. The sensitivity and specificity ratios of different cut-off values for D-dimer are also shown in Table 3. When determining the need for intensive care, the cut-off value for D-dimer was considered to be 1.0 mg/L. When patients with a D-dimer level of <1.0 mg/L were divided into two groups according to whether they needed intensive care or not, it was found that the mean MPV levels were not different between the groups (mean: 8.2 ± 1.1 vs. 8.3 ± 0.8, *p* = 0.73; Figure 3A). However, in patients with a D-dimer level of ≥1.0 mg/L, the mean MPV levels of patients who needed ICU transfer were significantly higher than those who did not need intensive care (8.6 ± 1.4 vs. 7.8 ± 0.9, *p* = 0.001) (Figure 3B). When D-dimer was <1.0 mg/L, reanalysis of ROC according to MPV revealed AUC: 0.550, 95% CI: 0.421–0.679 (Figure 4). On the other hand, the AUC for MPV was 0.694, 95% CI: 0.585–0.803, when D-dimer was ≥1.0 mg/L (Figure 5). In this case, different cut-off values for MPV are presented in Table 4. The cut-off value for MPV was considered to be 8.1. When patients with a D-dimer level of ≥1.0 mg/L were divided into two groups considering the MPV cut-off value as 8.1, the rate of ICU transport was determined to be significantly higher in patients with an MPV of ≥8.1 fL compared to those with an MPV of <8.1 fL (32.6 vs. 16.0%, *p* = 0.043; Table 5). Ultimately, for the prognostic efficacy of the combination of D-dimer ≥ 1.0 mg/L and MPV ≥ 8.1 fL in determining the need for intensive care, the values were determined as follows; sensitivity: 57.7%, specificity: 70.8%, positive predictive value (PPV): 32.0%, negative predictive value (NPV): 84.0%, accuracy: 63.0%.

**Table 3:** Cut-off values of D-dimer for intensive care unit admission state.

**D-Dimer**	**Sensitivity (%)**	**Spesificity (%)**
0.85	65.0	61.7
0.90	65.0	63.5
1.00	65.0	66.9
1.10	57.5	71.4
1.20	55.0	75.2

**Table 4:** Cut-off values of mean platelet volume when D-dimer ≥ 1.0 mg/L for intensive care unit admission state.

**MPV**	**Sensitivity (%)**	**Spesificity (%)**
7.25	92.3	33.7
7.75	80.8	50.6
8.05	57.7	65.2
8.15	57.7	70.8
8.45	46.2	74.2

*MPV, Mean platelet volume*.

**Table 5:** Comparison of intensive care unit admission rates according to mean platelet volume of 8.1 cut-off value when D-dimer ≥ 1.0.

**MPV**	**Not required** **ICU admission** ***n*, %**	**Required** **ICU admission** ***n*, %**	***p***
<8.1	58 (84.0)	11 (16.0)	0.043
≥8.1	31 (67.4)	15 (32.6)	

*ICU, intensive care unit; MPV, mean platelet volume*.

Regarding the secondary endpoints of our study, the duration of treatment and LOS, when D-dimer was ≥1.0, the median treatment duration in MPV < 8.1 and ≥8.1 groups was 5.0 (interquartile range: IQR: 5.0–10.0) days for both groups, and no difference was found (*p* = 0.64). The median duration of hospitalization was 7.0 (IQR: 5.0–10.5) days in the MPV < 8.1 group, while it was 8.5 (IQR: 5.0–16.3) days in the MPV ≥ 8.1 group, and no significant difference was found between them (*p* = 0.17).

## Discussion

The primary outcome of our study, the need for intensive care, was developed in nearly 1/8 of the hospitalized patients. ICU transfer was higher in male patients, those with symptoms of dyspnea, cough, and diarrhea at admission and in the presence of CAD and severe disease. Of the laboratory findings at the time of admission, lymphocyte and platelet counts were lower in patients who were admitted to the ICU, whereas inflammation markers, such as D-dimer and MPV parameters, were significantly higher. It was found that the prognostic efficacy of the MPV parameter was increased when combined with D-dimer and when the cut-off value was ≥8.1, it had moderate sensitivity and specificity, however, its negative predictive value was high. Albeit the duration of treatment and hospitalization were determined to be longer in the patients admitted to the ICU, no significant correlation was found between them and the marker generated by the combined use of these parameters.

Since the early stages of the pandemic, varying rates of ICU admissions have been reported across countries and regions all over the world. For instance, in a study involving the data of 1,099 patients in China, this rate was 6.1% (14) and in another study from China, it was 26% (3), while in the first months of the pandemic in the USA, 22% of 1,150 hospital admissions due to COVID-19 within a month were reported as critically ill (15). In a larger cohort of 5,700 patients, 14.2% of the patients required intensive care during follow-up (16). Consistent with the findings of our study, in many studies, advanced age, male gender, presence of comorbidities, such as hypertension, diabetes, cardiovascular diseases, decrease in laboratory parameters, such as lymphocyte and thrombocyte levels, increment in CRP, LDH, ferritin, D-dimer levels, have been reported at a high rate in patients transferred to ICU (3, 5, 6, 15–17). An increase in inflammation parameters, endothelial dysfunction, coagulation activation, and the thromboembolic event may occur particularly in patients who are hospitalized or require intensive care follow-up. D-dimer, which is a fibrin degradation product, is often used in determining the severity of host response in infectious diseases and risk assessment in sepsis and is one of the most studied markers among coagulation parameters in patients with COVID-19. In patients with infection or sepsis, high D-dimer levels were found to be associated with 28-day mortality (18). It was determined to be significantly higher in severe patients with COVID and in patients with mortality compared to other patients (19, 20). In a retrospective and multicentered study that included 191 patients, serum D-dimer > 1 μg/ml at admission was found to be an independent risk factor for in-hospital death (21). Furthermore, in another study, the median D-dimer value at the time of diagnosis was 1.0 mg/L in patients who applied to the emergency department with the suspicion of COVID-19 and whose disease was proven by PCR test (22). It has also been revealed that there is an increase in the D-dimer level as the disease severity increases, and an increase in the risk of mortality if it is >2 mg/L (23). Likewise, D-dimer levels were determined to be significantly higher in the 94 confirmed SARS-CoV-2-infected patient group compared to the healthy control group (24). In our study, upon assessing the efficacy of serum D-dimer level in indicating the need for ICU transfer by ROC analysis, we found that it had moderate sensitivity and specificity at a cut-off value of 1.0 mg/L.

Mean platelet volume, which is an inexpensive and user-friendly platelet indicator, has been investigated in various infectious and inflammatory conditions, such as septic shock. In COVID-19 disease, it is considered that the destruction of platelets by the immune system, bone marrow involvement, decrease in platelet count, and increased production of young platelets together with platelet activation induced by endothelial dysfunction lead to an increase in MPV. In a meta-analysis carried out by Lippi et al., thrombocytopenia was found to be associated with the severity of the disease (7). Of 383 patients with COVID-19, the mortality rate was higher in 68 patients with thrombocytopenia, whereas the rate of hospital discharge was lower (25). Consistent with this situation, we also found in our study that the median platelet counts were significantly lower, and the mean MPV values were high in patients who were transferred to the ICU at admission. In a study conducted in 506 patients with COVID-19 in India, MPV levels were determined to be significantly lower in those with moderate disease and those who survived, compared to the group with severe disease and mortality (26). In a prospective study that includes 46 patients in Italy, MPV levels were found to be higher in patients requiring mechanical ventilation support (27). In another study, in which 25 of 302 patients had died, the median MPV values were determined to be significantly higher in patients with mortality (28). In the study in which platelet reactivity was investigated and 60 patients with COVID-19 and 60 healthy control groups were compared, the median MPV values were 10.5 and 9.8 fL, respectively, and the difference between the two groups was found to be significant (29). Moreover, Barrett et al. found the median MPV levels to be higher in patients with COVID-19 who developed thrombosis or death compared to patients who did not (11.0 vs. 10.5, *p* = 0.022) (30). It is noticed that the values reported for MPV levels in the literature vary. We are of the opinion that the heterogeneity of the patient groups in the studies and the different disease severities, the retrospective data obtained in most studies, and the differences due to study designs play a considerable role in these varying results. In this study, we sought to determine a combined parameter and cut-off value to predict poor prognosis in patients with conditions requiring hospitalization. It was found out that the efficacy of MPV alone or when D-dimer was <1.0 mg/L was weaker in terms of determining ICU transfer, whereas its prognostic efficacy was increased when D-dimer was ≥1.0 mg/L. When the cut-off value was considered to be 8.1 fL for MPV, it was determined that the need for intensive care was significantly higher at this level and over. While the sensitivity and specificity of the combination of D-dimer ≥ 1.0 mg/L and MPV ≥ 8.1 fL in predicting the need for intensive care were moderate, we found it low for PPV but high for NPV. Yet, we did not find a correlation between this combined parameter and the duration of treatment and hospitalization of the patients. In a study that includes 85 patients, it was revealed that MPV/platelet ratio was associated with the serious disease; however, no difference was found between the groups regarding the duration of hospitalization (31). In a study in which the mean hospital stay was 6.3 days when patients were categorized based on a hospitalization period of ≤ 1 week and >1 week, no difference was found between the groups in terms of platelet and MPV values (32). Similarly, in our patient cohort, the median LOS was longer (7.0 vs. 8.5 days) in the group with MPV ≥ 8.1 fL compared to the group with MPV < 8.1 fL, but the difference between the two groups was not significant.

The fact that our study was single-centered and included patient data from a hospital providing tertiary care constitutes the main limitation regarding the generalization of the findings. Besides, we consider that due to its retrospective design, the risk of selection bias in the case of ICU transfer should be taken into account. For the same reason and regarding methods of the study, other circumstances that cause high MPV levels could not be a detailed review. Diagnosis of COVID-19 was based on PCR results and/or the presence of clinical and radiological features of the disease. If the NP swab test was negative, the patient was excluded. NP swab test was negative but there were typical radiological and clinical findings for COVID-19 also started with specific therapy for COVID-19, we included these cases in the study. Another point, in patients who needed ICU admission, D-dimer values were significantly higher than patients who did not need. Although possible PE might be discussed, patients with PE proved with objective methods were excluded at the beginning of the study. Albeit standard treatment has been provided to all patients in our hospital since the beginning of the pandemic, in accordance with the guidelines, it can be expected that different treatment approaches between centers and countries would also impact the outcomes. However, we think that MPV, which is an easy-to-reach, fast, safe, and inexpensive parameter, deserves to be investigated as a biomarker in the struggle against the disease under pandemic conditions, and in this regard, we consider that our study can contribute to the knowledge of the literature.

In conclusion, it was found out that a minimum 2-fold increase in serum D-dimer level and an MPV value of ≥8.1 fL in patients with COVID-19, who were hospitalized and had bilateral infiltration on chest tomography, were found to have moderate efficacy in determining the need for intensive care and that it has a relatively higher NPV. This combined parameter may be helpful for determining the need for ICU transfer and further prospective researches need to be done. Yet, no correlation was found between the combined use of these parameters, regarding the duration of treatment and hospitalization.

## Data Availability Statement

The raw data supporting the conclusions of this article will be made available by the authors, without undue reservation.

## Ethics Statement

The studies involving human participants were reviewed and approved by Ethics Committee of the University of Health Sciences Sureyyapasa Chest Diseases and Thoracic Surgery Training and Research Hospital (Date: 04.06.2020, Protocol code: 116.2017.161). Written informed consent for participation was not required for this study in accordance with the national legislation and the institutional requirements.

## Author Contributions

ND contributed to study design, analysis and interpretation of data, preparing the manuscript, and final approval of the version to be published. OO contributed to the conception of the work, collection and analysis of data, and revise the text finally. SBo contributed to the study design, collection and interpretation of data, and approve the work in ensuring that appropriately investigated. CA and MA contributed to the collection and analysis of data, prepare the manuscript, and review of the final text. MK contributed to find research questions, study design, manuscript preparation, editing, and review of the manuscript. SAy contributed to the analysis of data, search of literature, and preparation of the manuscript. BG contributed to study design, interpretation of data, and preparation and review of the manuscript. OS contributed to data collection, data analysis, review, and final approval of the manuscript. SBe contributed to find research question, analysis of data, and preparation of the manuscript. AO contributed to the conception of the work, collection of data, and revise the text finally. SAr contributed to the search of literature, data collection, and preparation of the manuscript. DD contributed to study design, interpretation of data, and approve the work in final version. HT contributed to the analysis of data, search of literature, and final approval of the manuscript. FY and DT contributed to data collection, data analysis, and review of the manuscript. FO contributed to find research question, study design, and preparation of the manuscript. ES contributed to collection and analysis of data, prepare the manuscript, and review of the final text. EB contributed to search of literature, data collection, and final approval of the manuscript. LD contributed to study design, manuscript preparation, editing, and review of the manuscript. UA contributed to data collection, analysis of data, and preparation of the manuscript. SO contributed to develop research question, collection of data, and final approval of the manuscript. DE contributed to the conception of the work, data analysis, and revise the text finally. GG contributed to data analysis, preparation of the manuscript, and final approval of the manuscript. NA contributed to the search of literature, analysis of data, and preparation of the manuscript. TY contributed to find research question, editing, and review of the manuscript. OM contributed to study design, interpretation of data, and review of the manuscript. HG contributed to the conception of the work, collection of data, and final approval of the text. RY contributed to literature search, data collection, and editing of the manuscript. TS contributed to collection, analysis of data, and manuscript preparation. TT contributed to study design, data collection, and review of the manuscript. All authors contributed to the article and approved the submitted version.

## Conflict of Interest

The authors declare that the research was conducted in the absence of any commercial or financial relationships that could be construed as a potential conflict of interest.

## Publisher's Note

All claims expressed in this article are solely those of the authors and do not necessarily represent those of their affiliated organizations, or those of the publisher, the editors and the reviewers. Any product that may be evaluated in this article, or claim that may be made by its manufacturer, is not guaranteed or endorsed by the publisher.
